# Development and Validation of a Prognostic Nomogram Based on DNA Methylation-Driven Genes for Patients With Ovarian Cancer

**DOI:** 10.3389/fgene.2021.675197

**Published:** 2021-09-09

**Authors:** Min Zhou, Shasha Hong, Bingshu Li, Cheng Liu, Ming Hu, Jie Min, Jianming Tang, Li Hong

**Affiliations:** Department of Gynecology and Obstetrics, Renmin Hospital of Wuhan University, Wuhan, Hubei, China

**Keywords:** ovarian cancer, methylation, CpG sites, model, overall survival, biomarkers

## Abstract

**Background:** DNA methylation affects the development, progression, and prognosis of various cancers. This study aimed to identify DNA methylated-differentially expressed genes (DEGs) and develop a methylation-driven gene model to evaluate the prognosis of ovarian cancer (OC).

**Methods:** DNA methylation and mRNA expression profiles of OC patients were downloaded from The Cancer Genome Atlas, Genotype-Tissue Expression, and Gene Expression Omnibus databases. We used the R package *MethylMix* to identify DNA methylation-regulated DEGs and built a prognostic signature using LASSO Cox regression. A quantitative nomogram was then drawn based on the risk score and clinicopathological features.

**Results:** We identified 56 methylation-related DEGs and constructed a prognostic risk signature with four genes according to the LASSO Cox regression algorithm. A higher risk score not only predicted poor prognosis, but also was an independent poor prognostic indicator, which was validated by receiver operating characteristic (ROC) curves and the validation cohort. A nomogram consisting of the risk score, age, FIGO stage, and tumor status was generated to predict 3- and 5-year overall survival (OS) in the training cohort. The joint survival analysis of DNA methylation and mRNA expression demonstrated that the two genes may serve as independent prognostic biomarkers for OS in OC.

**Conclusion:** The established qualitative risk score model was found to be robust for evaluating individualized prognosis of OC and in guiding therapy.

## Introduction

Ovarian cancer (OC), the most lethal gynecological cancer, is the seventh most common cancer and the fifth leading cause of cancer-related deaths in women, with a 5-year survival rate of 47.4% (Howlader et al., 2019). In the United States, over 22,000 new cases are diagnosed, and 14,000 patients die each year (Siegel et al., 2020). OC is a highly fatal malignancy with an insidious onset, and there is currently a lack of a definitive screening tool and diagnosis often occurs only at later stages. Most OCs originate from the epithelium, and surgery and cytoreduction are used as the main forms of treatment, followed by combined chemotherapy. Although progress has been made, curative and survival trends have not changed significantly. In addition, OC is heterogeneous and often prone to developing chemotherapy resistance (Cho and Shih, 2009). Therefore, exploring the pathogenesis of OC, formulating effective methods of early screening and diagnosis, and finding new prognostic biomarkers and treatment pathways for OC would help improve the therapeutic effect and survival rate of patients with OC.

DNA methylation, one of the main epigenetic alterations, has an important impact on the initiation and progression of cancer (Jones and Baylin, 2007). DNA hypermethylation silences gene expression by adding a methyl group to the promoter region of DNA, thereby regulating gene expression (Nervi et al., 2015). At the same time, cancer can promote global hypermethylation of CpG islands related to the promoter, thereby silencing important genes for cell homeostasis, such as tumor suppressor genes (TSGs). Moreover, demethylation mainly involves chromosomal instability, reactivation of transposons, and loss of genomic imprinting (Deng et al., 2020). Conversely, demethylation can promote expression of oncogenes. Abnormal DNA methylation and changes in chromatin structure can alter gene expression and promote tumorigenesis. Therefore, methylation-regulated gene expression, including oncogene and TSGs, plays a double-sided role in EOC development.

Dysregulation of DNA methylation has also been observed in OC (Ishak et al., 2019). Hypermethylation is associated with the inactivation of almost all pathways involved in the occurrence and development of EOC, such as DNA repair, cell apoptosis, and adhesion (Makarla et al., 2005; Natanzon et al., 2018). The promoters of certain tumor suppressors, such as ZNF671, BRCA1, and RASSF1A, are hypermethylated in OC as compared to those in non-neoplastic tissues (Ibanez de Caceres et al., 2004; Mase et al., 2019). It is generally believed that hypermethylation of TSGs or hypomethylation of oncogenes is an important mechanism of tumorigenesis (Pan et al., 2018). Furthermore, DNA methylation changes in circulating blood can be used to detect and predict early-stage OC (Barton et al., 2008). Numerous studies have performed a comprehensive multi-omics analysis of EOC genomics, epigenomics, and transcriptomics, and suggested that gene methylation plays an important role in OC development (Zheng et al., 2019). Hence, a comprehensive analyses of DNA methylation and mRNA expression are essential for understanding the biological processes of OC. In addition, only a few methylation markers of OC have been widely accepted and are being applied in clinical practice.

At present, numerous studies have used the R package *MethylMix* to screen methylation-driven genes, explore possible markers, establish related models, and predict the correlation between the target gene methylation level and diagnosis, survival, and recurrence of malignant tumors, which can help clarify the occurrence and development of malignant tumors (Bai et al., 2020; Peng et al., 2020; Zhang et al., 2020). In this study, we obtained DNA methylation and mRNA expression profiles of OC patients from The Cancer Genome Atlas (TCGA), Genotype-Tissue Expression (GTEx), and Gene Expression Omnibus (GEO) databases. Then, we used the R package *MethylMix* to identify DNA methylation-regulated DEGs and built a prognostic signature using LASSO Cox regression analysis. Then, a quantitative nomogram was drawn based on the risk score and clinicopathological features, and the predictive ability of the signature was confirmed in different datasets.

## Materials and Methods

### Dataset Acquisition and Pre-processing

The DNA methylation data of OC were downloaded from TCGA^1^ database. The mRNA expression profiles of normal ovarian and OC samples were downloaded from the GTEx and TCGA databases using the University of California Santa Cruz (UCSC) Xena browser (Chang et al., 2019). In addition, the microarray data of GSE9891 and GSE26712 were acquired from GEO^2^ to represent independent cohorts of OC. Patients without survival time or status were excluded from the study. To ensure that the established prognostic signature had better generalization, TCGA dataset was used as the training set, and GSE9891 and GSE26712 datasets were used as the validation set. Cases without a certain age, FIGO stage, and tumor grade were excluded. Finally, 358 OC patients were included in TCGA set, 273 patients in the GSE9891 set, and 185 patients in the GSE26712 set. Table 1 lists the clinical features of the patients in the training and validation sets.

**TABLE 1:** Clinicopathologic characteristics of ovarian cancer (OC) patients in The Cancer Genome Atlas (TCGA) and Gene Expression Omnibus (GEO) cohorts.

Variables	TCGA cohort	GSE9891
	(*n* = 358)	(*n* = 273)
	*N* (%)	*N* (%)
Age (M ± SD, years)	59.4 ± 11.39	59.60 ± 10.55
Tumor size (M ± SD, cm)	0.90 ± 0.40	–
Grade		
1 and 2	43 (12.0)	112 (41.1)
3 and 4	315 (88.0)	161 (58.9)
Tumor status		
Tumor free	80 (22.3)	–
With tumor	236 (65.8)	–
Unknown	42 (11.7)	–
Lymphatic invasion		
No	46 (12.8)	
Yes	97 (27.1)	
Unknown	215 (60.1)	
Venous invasion		
No	38 (10.6)	–
Yes	60 (16.8)	–
Unknown	260 (72.6)	–
Stage		
I–II	20 (5.6)	41 (15.0)
III	284 (79.3)	209 (76.6)
IV	54 (15.1)	23 (8.4)
Primary therapy outcome		
Complete remission/response	202 (56.4)	–
Partial remission/response	42 (11.7)	–
Stable disease	21 (5.9)	–
Progressive disease	25 (7.0)	–
Unknown	68 (19.0)	–

### Identification of Methylation-Related DEGs

The differential expression analysis was conducted using the R package *limma*. The criteria for identifying DEGs were |Log_2_ (fold change)| >  0.585 and *P* value < 0.05, and the criteria for identifying differentially methylated genes (DMGs) were |Log_2_ (fold change)| >  0 and adjusted *P* value < 0.05. Intersecting genes of DMGs and DEGs that had significantly different methylation levels and expression levels were retained. Pearson correlation coefficients between the methylation and expression levels of the intersecting genes were calculated using the *MethylMix* package in R (Pan et al., 2019). Intersecting genes with negative coefficients were used for the subsequent analyses. Pearson coefficient < −0.3 and *P* < 0.05 were set as criteria for identifying methylation-regulated DEGs.

### Construction of Prognostic Risk Model

We conducted further analyses to determine the survival significance of methylation-regulated DEGs. Using *P* < 0.05 as the cutoff value, we conducted univariate Cox proportional hazard regression analysis for DEGs in the training set. Prognosis-related genes constructing a prognostic risk model in OC were further analyzed and selected by LASSO-penalized Cox regression analysis. Finally, a prognostic signature was built with the expression levels of methylation-regulated DEGs and their corresponding coefficients, as shown below.


$$\mathrm{Risk}\,\mathrm{score}=\sum_{i\,=1}^{N}\mathrm{Exp}\times \text{Coef}.$$

where Exp represents the expression value of each methylation-regulated DEG and Coef represents the regression coefficient. All samples were split into two subgroups using the median value: high- and low-risk. The distribution of patients with different risk scores was evaluated using principal component analysis (PCA). Kaplan–Meier (KM) survival curves were plotted to assess the difference in OS between the two subgroups, and receiver operating characteristic (ROC) curves were used to evaluate the accuracy of the model with area under curve (AUC) values. In addition, two independent GEO datasets (GSE26712 and GSE9891) were used to validate the performance of the signature.

### Construction of Prognostic Nomogram

To explore whether the prognostic signature could be independent of other clinical variables (including age, tumor size, tumor status, and tumor stage), we conducted univariate and multivariate Cox regression analyses. A prognostic nomogram for OC patients was constructed based on the risk score and other independent prognostic parameters. The distinguishing ability of the nomogram was assessed using AUC. The calibration curves were plotted to compare the nomogram-predicted survival with the actual survival.

### Joint Survival Analysis of DMGs and DEGs

We conducted a joint survival analysis of DNA methylation and mRNA expression levels of the same methylation-regulated gene to further identify key genes associated with prognosis in patients with OC.

### Functional Enrichment Analysis

Functional enrichment analyses were conducted to explore the potential molecular mechanisms underlying the prognostic signatures. We used the R package *limma* to detect differentially expressed genes (DEGs) between the high- and low-risk groups (|Log_2_FC | >  1 and FDR <  0.05). Gene Ontology (GO) and Kyoto Encyclopedia of Genes and Genomes (KEGG) pathway enrichment analyses of the differentially expressed FRGs were analyzed using the R package *ClusterProfiler* by setting *p* < 0.05 and *q* < 0.05.

### Statistical Analysis

The predictive ability of the prognostic model was evaluated using the AUC values of the ROC curves. PCA was conducted using the “prcomp” function of the R package *stats*. A nomogram comprising the risk score and clinical variables was built to predict the 3- and 5-year OS using the *rms* package. All statistical analyses were performed using the R software (Version 3.5.3).

## Results

### Identification of Methylation Related DEGs

**FIGURE 1:**
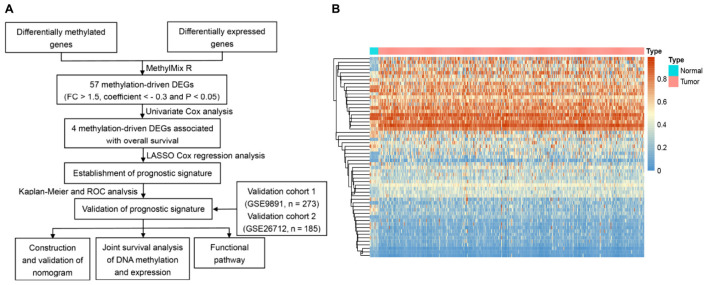
Identification of methylated related genes and flowchart of the establishment of novel prognostic signature. **(A)** The flowchart of the establishment of novel prognostic risk model for patients with ovarian cancer (OC). **(B)** The heatmap plot of 57 methylation related differentially expressed genes (DEGs) in OC. The color change from blue to red in the heatmap illustrates the trend from low to high methylation.

The entire data processing flow is shown in Figure 1A. According to the screening criteria (| FC| > 1.5 and FDR < 0.01), we confirmed that the expression of 2,391 genes, with 1,195 up-regulated and 1,196 down-regulated genes, was significantly different between the normal control group and OC group. After calculating the Pearson correlation coefficients between the methylation and expression levels, 57 genes with negative correlation coefficients were identified as methylation-related DEGs (Figure 1B).

### Construction and Validation of Prognostic Signature

**FIGURE 2:**
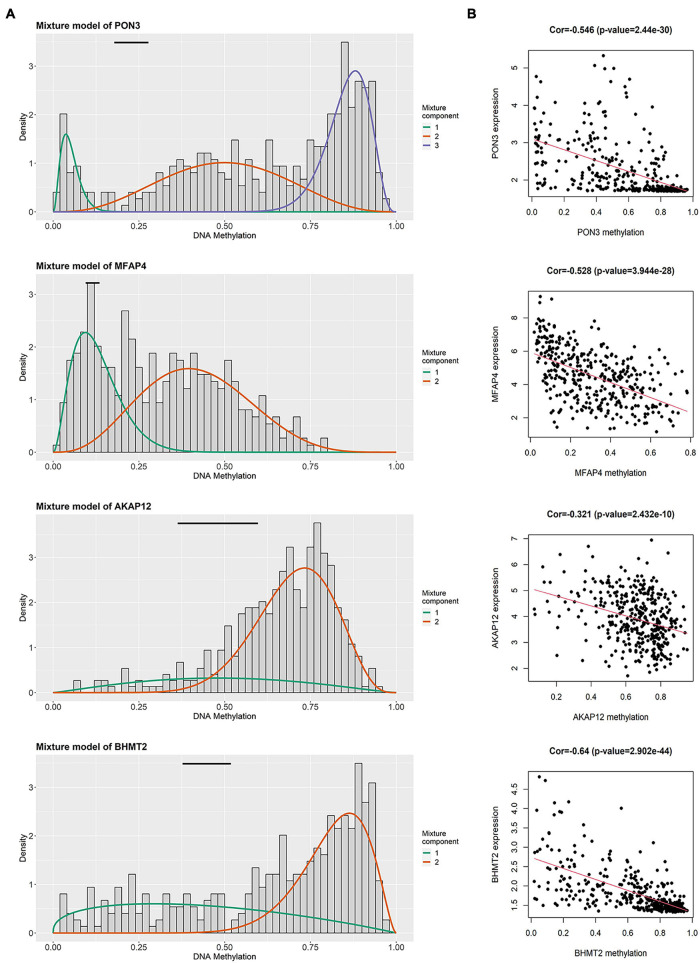
Summary of four methylated related genes in the signature. **(A)** The distribution map of methylated status of four genes. The histogram demonstrates the distribution of methylation in tumor samples. Horizontal black bars show the distribution of methylation in normal samples. **(B)** The correlation between DNA methylation level and mRNA expression level in four genes.

**FIGURE 3:**
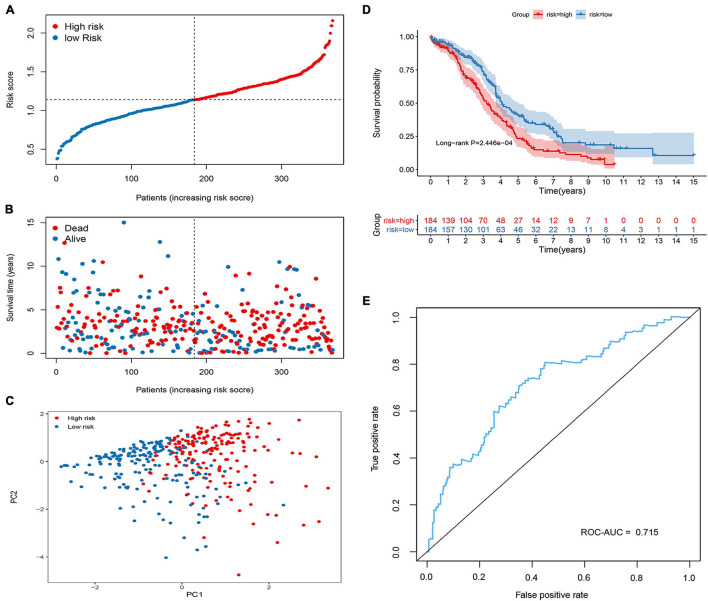
Construction of the methylated related gene signature in the Cancer Genome Atlas (TCGA) cohort. **(A)** Dot plot of risk score. *Y* axis represents risk score. Red and blue color dots represent, respectively, high and low risk score samples. **(B)** Dot plot of survival. *Y* axis represents survival times (years). Red and blue color dots represent, respectively, dead and living OC samples. **(C)** PCA plot of the TCGA cohort. **(D)** Kaplan–Meier (KM) estimate of the overall survival (OS) in the TCGA cohort. **(E)** The time-dependent ROC curves in the TCGA cohort.

In the training group, four prognosis-related genes were selected using univariate Cox regression, and the optimal gene combination was identified using the Lasso Cox regression model. Finally, a signature consisting of four methylation-related DEGs was built as a prognostic model for patients with OC. *Risk score = (−0.164 × expression level of PON3) + (0.0559 × expression level of MFAP4) + (0.1779 × expression level of AKAP12) + (0.3056 × expression level of BHMT2)*. *All the four methylation-related DEGs* were hypermethylated (Figure 2A). As shown in Figure 2B, there was a significant negative correlation between DNA methylation and gene expression levels. The distribution of risk scores and the relationship between risk scores and survival time of OC patients were visually analyzed (Figures 3A,B). Using the median score value, we divided all patients into two sub-populations, namely high- and low-risk groups. PCA demonstrated that the patients in the different risk groups were distributed in two directions (Figure 3C). The KM curves were plotted in the training cohort according to the risk score, and the high-risk group showed a poor OS compared to the low-risk group (Figure 3D). The tdROC curves revealed that the prognostic signature had superior predictive accuracy, with an AUC of 0.715 in the training set (Figure 3E). We then conducted a subgroup-level analysis of the OS of patients with different ages, grades, tumor sizes, tumor status, and FIGO stages. The results revealed that in subtype age, tumor status, and FIGO staging, the OS of OC patients with high-risk scores was shorter than that of OC patients with low-risk scores (Supplementary Figure 1).

### External Validation of the Prognostic Signature

**FIGURE 4:**
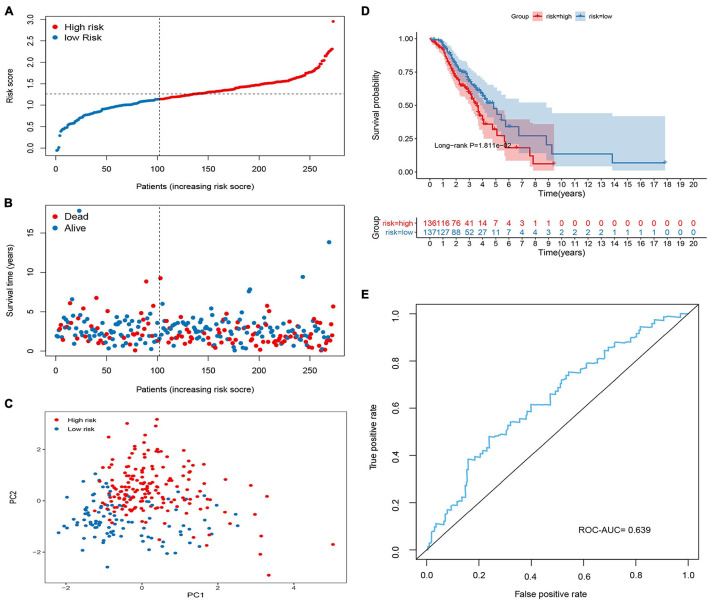
Validation of the methylated related gene signature in the GSE9891 cohort. **(A)** Dot plot of risk score. *Y* axis represents risk score. Red and blue color dots represent, respectively, high and low risk score samples. **(B)** Dot plot of survival. *Y* axis represents survival times (years). Red and blue color dots represent, respectively, dead and living OC samples. **(C)** PCA plot of the GSE9891 cohort. **(D)** KM estimate of the OS in the GSE9891 cohort. **(E)** The time-dependent ROC curves in the GSE9891 cohort.

**FIGURE 5:**
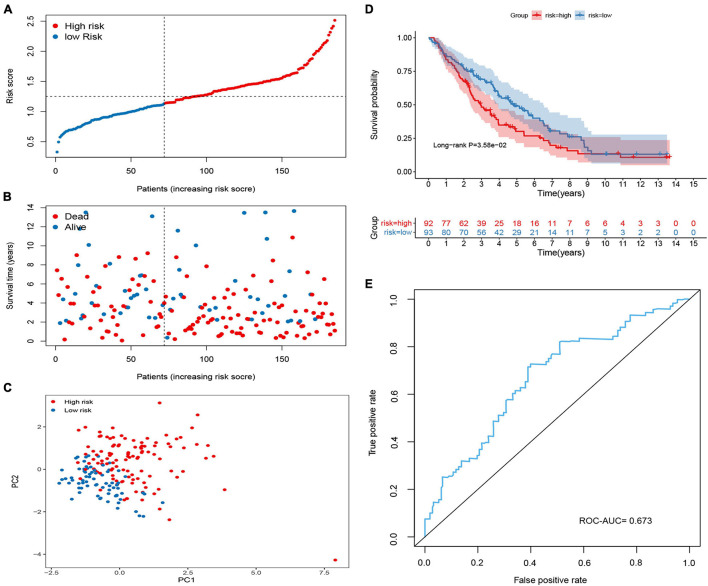
Validation of the methylated related gene signature in the GSE26712 cohort. **(A)** Dot plot of risk score. *Y* axis represents risk score. Red and blue color dots represent, respectively, high and low risk score samples. **(B)** Dot plot of survival. *Y* axis represents survival times (years). Red and blue color dots represent, respectively, dead and living OC samples. **(C)** PCA plot of the GSE26712 cohort. **(D)** KM estimate of the OS in the GSE26712 cohort. **(E)** The time-dependent ROC curves in the GSE26712 cohort.

To validate the predictive ability of the signature in predicting OS, the risk score of each patient was calculated in two independent sets (GSE26712 and GSE9891; Figures 4, 5). The distribution of risk scores, the relationship between risk scores and survival time of OC patients, and PCA for the two independent sets are shown in Figures 4A–C, 5A–C. Consistent with the results in the training set, the KM analysis indicated that high-risk patients showed poorer OS (Figures 4D, 5D). The ROC curve showed that the signature had good accuracy, with AUC values of 0.639 and 0.673, respectively (Figures 4E, 5E).

### Construction of Prognostic Nomogram

**FIGURE 6:**
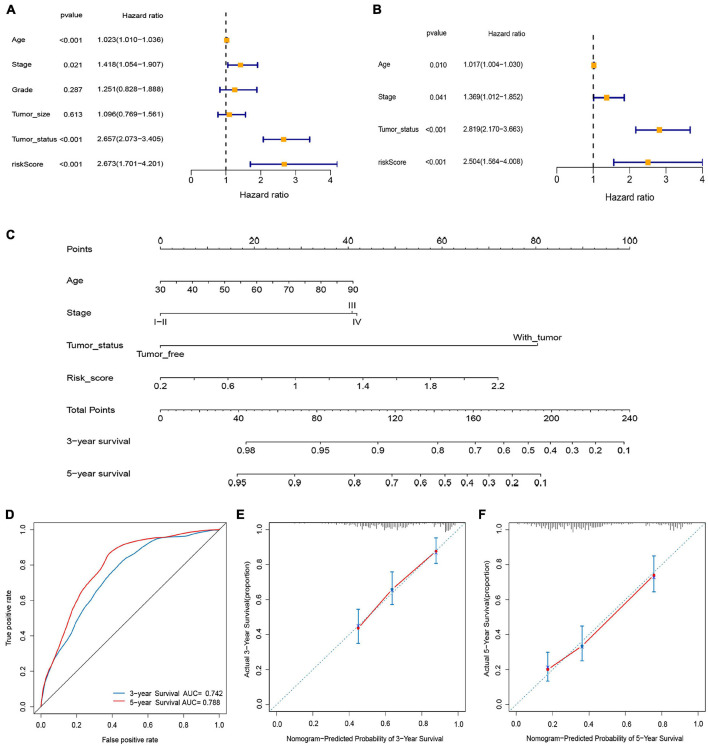
Construction and validation of nomogram. **(A)** Univariate Cox analysis. **(B)** Multivariate Cox analysis. **(C)** Nomogram for predicting 3- and 5-year overall survival of OC patients. **(D)** Time-dependent ROCs for 3- and 5-year OS of the nomogram. **(E,F)** Calibration curves of the nomogram prediction of 3-and 5-year OS of patients with OC.

Univariate Cox regression analysis demonstrated that age, stage, tumor status, and risk score were associated with the prognosis of OC patients (Figure 6A, *P* < 0.05), and were confirmed as independent predictors of OS using the multivariate Cox regression analysis (Figure 6B, *P* < 0.05). We then established a prognostic nomogram by quantifying these clinical factors (Figure 6C). The AUCs of the nomogram predicting the 3- and 5-year OS rates were 0.742 and 0.788, respectively (Figure 6D). The calibration curves demonstrated that the predicted 3- and 5-year OS rates closely corresponded with the actual survival rates with a 10% error margin, represented by the dotted lines (Figures 6E,F).

### Joint Survival Analysis

The joint survival analysis demonstrated that the combination of methylation and expression of the two genes was correlated with patient prognosis (*P* < 0.05). The OS associated with hypomethylation and high expression of *CIDEB* and *SLC52A3* was lower (Supplementary Figure 2).

### Functional Enrichment Analysis

Gene Ontology functional enrichment analysis revealed that the DEGs between the high- and low-risk groups were enriched in immune-related biological processes, including humoral immune response mediated by circulating immunoglobulin, regulation of humoral immune response, and B cell-mediated immunity (Supplementary Figure 3A). The KEGG pathway revealed that the differential gene pathway included cell adhesion molecules, cell cycle, Ras signaling pathway, and Th1 and Th2 cell differentiation (Supplementary Figure 3B).

## Discussion

The OC is the most lethal gynecological cancer with a poor prognosis. It is insidious, difficult to diagnose early, and prone to relapse and developing chemoresistance. In addition, the clinical outcomes of advanced OC remain unsatisfactory (Lheureux et al., 2019). Thus, there is a need to formulate a new quantitative prediction method to accurately assess the prognosis of patients with OC. In the present study, the methylation and gene expression data of OC samples were analyzed to construct a qualitative risk score system for predicting the OS of patients with OC. A total of 56 methylation-related DEGs were obtained, and a prognostic signature with four genes was constructed in the training set. We validated its predictive accuracy in two independent GEO sets and confirmed that the model was reliable, regardless of the dataset used in the independent validation set. The OS of patients with high-risk scores was poorer than that of patients with low-risk scores. Subgroup analysis revealed that the subgroups based on age, stage, and tumor status were equally meaningful. Multivariate analysis indicated that the model could be an independent risk predictor of OC. We then constructed a quantitative nomogram that integrated the risk score and clinical features. The joint survival analysis revealed that the combination of methylation and expression of two genes is an independent predictor of OS in OC. Hence, the signature not only served as an independent risk predictor of OC, but also helped identify high-risk patients and guide individualized treatment. This has significance in clinical applications.

Our study has several advantages. The high-throughput “omics” data combined with bioinformatic analysis provided valid and economical methods to depict the prognostic value of model. Owing to the clinical heterogeneity among OC patients, single prognostic biomarkers could not be enough to accurately predict the patient’s prognosis. Integrating multiple biomarkers into a single prediction model may maximize the advantages of single biomarkers and the accuracy of prognostic prediction value across data sets. In present study, we integrated methylomes and transcriptomes profiles to identify methylation-related DEGs, and integrated multiple biomarkers into a single model that would substantially improve prognostic value compared with a single biomarker. This model is found to be good in two validated cohorts. In many studies, inappropriate statistical methods were used to mine microarray data. In the planning of survival analysis to model covariate information, Cox proportional hazards regression analysis is the most popular method; however, it is not suitable for high-dimensional microarray data when the ratio of sample size to variable is too low. LASSO Cox regression analysis can perform dimensional analysis more effectively to construct more accurate genetic (Ternès et al., 2016). The lambda value with the minimum average error obtained from the cross-validation method was fitted into the LASSO regression analysis to filter genes. In present study, we used LASSO Cox regression to select markers in the prognostic signature. Therefore, the predictive ability of the signature is more reliable and accurate. Furthermore, the nomogram combining risk score and clinical parameters can provide a visual method for predicting individual OS in OC patients.

Aberrant DNA methylation in the promoter region is usually considered a hallmark of tumors, which usually leads to the abnormal activation of oncogenes and the transcriptional silencing of TSGs (Cruickshanks et al., 2013). Studies have shown that aberrant DNA methylation often occurs in early tumors and that epigenetic changes are relatively stable (Ibrahim et al., 2011). The methylation profile of the gene promoter varies with cancer type, which indicates that the detection of aberrant methylation may serve as a potential molecular biomarker for cancers. In addition, as epigenetic changes are reversible, they are expected to be therapeutic targets (Baylin and Jones, 2011). Therefore, dysregulation of DNA methylation may serve as a biomarker for clinical diagnostic and prognostic evaluation, and clinical decision-making of tumors.

Aberrant DNA methylation has been reported to influence the development and progression of OC. Dysregulated DNA methylation-related genes can promote malignant transformation through the silencing of TSGs or overexpression of oncogenes, which constitutes a new balance in the tumor microenvironment and may become a predictive biomarker of prognosis. TSGs usually show promoter hypermethylation and inhibit its expression, thereby promoting the pathogenesis of OC (Chen et al., 2018; Rezk et al., 2018). The promoter of TSGs (*BRCA1* and *RASSF1A*) is hypermethylated in OC tissues (Ibanez de Caceres et al., 2004). Hypermethylation silences expression to inhibit *BRCA1* function, driving genomic instability in OC (Rezk et al., 2018). Silencing of *RASSF1A* promotes cell cycle progression and uncontrolled cell growth. Deng et al. (2017) revealed that expression of the tumor suppressor *miR-199a-3p* was significantly down-regulated in OC cells, and its promoter was hypermethylated in OC cells. Overexpression of miR-199a-3p can inhibit the migration, invasion, and tumorigenic capabilities of OC cells as well as enhance cisplatin resistance by inhibiting targeted DDR1 expression. Similarly, expression of the *miR-424/503* cluster is inhibited by DNA hypermethylation in the promoter regions, which promotes the expression of *KIF23*, thereby improving the oncogenic performance of OC cells (Li et al., 2019).

Among the four methylation-driven DEGs, paraoxonase 3 (*PON3*), a member of the lipolactonases family, regulates mitochondrial function and reduces the release of superoxide anion free radicals in the inner mitochondrial membrane (Schweikert et al., 2012). In addition to its antioxidant effect, *PON3* may also have an anti-apoptotic effect, which may be related to the physiology and pathology of tumor cells (Witte et al., 2012). Promoter hypermethylation of *PON3* and/or decreased mRNA expression has been reported in several types of cancers, including OC (Kitchen et al., 2016; Shui et al., 2016; Wouters et al., 2017; Song et al., 2020). Song et al. (2020) used reduced representation bisulfite sequencing to investigate OC-specific DNA methylation and gene expression in 21 OC tissues and adjacent normal tissues, and revealed that 11 differentially methylated regions in *CAPS*, *FZD7*, *CDKN2A*, *PON3*, and *KLF4* genes were significantly hypermethylated and down-regulated. They then confirmed their methylated levels in another 41 pairs of OC tumors and normal tissues. *PON3* was down-regulation and hypermethylated in the TP53 mutant OC. Similar patterns of epigenetic regulation have also been reported in DNA methylation studies of several other cancers (Baharudin et al., 2017; Huang et al., 2018), indicating that *PON3* may be a tumor suppressor in OC. Shui et al. (2016) identified two differentially methylated regions in the gene *PON3*, whose promoter hypermethylation was correlated with decreased mRNA expression. Reduced *PON3* expression in the presence of promoter methylation was confirmed in another study (Kitchen et al., 2016). Microfibrillar-associated protein 4 (MFAP4), known as 36-kDa microfibril-associated glycoprotein (MAGP36), is ubiquitously distributed in the extracellular matrix of the human body (Toyoshima et al., 2005; Schlosser et al., 2006). MFAP4 has been associated with immune response (Niu et al., 2011), liver fibrosis (Madsen et al., 2020), renal fibrosis (Pan et al., 2020), atherosclerosis (Wulf-Johansson et al., 2013), pulmonary airspace enlargement (Holm et al., 2015), and abdominal aortic aneurysms (Lindholt et al., 2020). MFAP4 was also found to be involved in human cancers, such as pancreatic adenocarcinoma (Guerrero et al., 2021), serous OC (Zhao et al., 2019), breast cancer (Yang et al., 2019), and lung cancer (Feng et al., 2020). Yang et al. (2019) found that MFAP4 was down-regulated and may function as a tumor suppressor in breast cancer. Elevated MFAP4 levels are associated with better overall survival (OS). Promoter hypermethylation of MFAP4 results in the down-regulation of its mRNA expression. Similarly, a significant negative correlation between DNA methylation values and mRNA expression of MFAP4 was observed in serous OC (Zhao et al., 2019). Patients with high MFAP4 levels were associated with poorer OS and recurrence-free survival. This finding was consistent with our results. A kinase anchoring protein 12 (AKAP12) is a scaffolding protein that can bind to protein kinase A and protein kinase C to regulate signal transduction (Gelman, 2012; Wu et al., 2018). AKAP12 can also control cell adhesion, mitogenesis, and differentiation and has tumor suppressing properties. AKAP12 promoter CpG island hypermethylation and low expression have been reported in various cancers, including colorectal cancer (Mori et al., 2006), juvenile myelomonocytic leukemia (Wilhelm et al., 2016), and prostate cancer (Gelman, 2012). 5-aza-2′-deoxycytidine can reverse AKAP12 promoter hypermethylation and restore AKAP12 expression. Wilhelm et al. (2016) found that the AKAP12α promoter was hypermethylated in juvenile myelomonocytic leukemia tissues, which was associated with decreased AKAP12α expression. Hypermethylation of the AKAP12α promoter is linked to delayed diagnosis, elevated levels of fetal hemoglobin, and poor prognosis. Qian et al. (2020) indicated that AKAP12 was down-regulated in myelodysplastic syndrome tissues, and up-regulation of AKAP12 prolonged the cell cycle, inhibited cell proliferation, and induced apoptosis by activating the ERK1/2 signaling pathway. *BHMT2* was reported to be down-regulated in hepatocellular carcinoma tissues compared with adjacent normal tissues (Pellanda et al., 2012), but it has not been reported to be related to OC biology.

This study has some limitations that require further research. First, although the signature based on methylation-driven genes has been validated in TCGA dataset and the two GEO datasets using different technology platforms, more independent datasets are still needed to verify the signature to ensure its robustness and repeatability. In addition, the signature was established using our own computational framework. Therefore, further functional studies are required to validate our results. Second, although the nomogram incorporates age, stage, tumor status, and risk score to predict the OS rates, the clinicopathological variables were considered insufficient due to limited data. Third, although our quantitative prognostic model is promising, it is too early to assert that our two-dimensional model (epigenetic and transcriptional signatures) is superior to traditional examinations.

In conclusion, we established a prognostic risk model consisting of four methylation-driven genes in OC and validated the results using different datasets. We also confirmed that the signature as an independent predictor was significantly associated with prognosis. The nomogram integrating the risk score and clinicopathological features was found to be robust in predicting the OS of patients with OC. The qualitative model described herein may serve as a reliable and reproducible tool for prognostic prediction in individual cases.

## Data Availability Statement

The original contributions presented in the study are included in the article/Supplementary Material, further inquiries can be directed to the corresponding author.

## Ethics Statement

Ethical review and approval was not required for the study on human participants in accordance with the local legislation and institutional requirements. Written informed consent for participation was not required for this study in accordance with the national legislation and the institutional requirements. Written informed consent was not obtained from the individual(s) for the publication of any potentially identifiable images or data included in this article.

## Author Contributions

MZ and LH designed the research study. MZ, BL, and CL performed the research. MZ, BL, SH, and MH analyzed the data. MZ and BL wrote the manuscript. MZ and SH revised the manuscript. All authors contributed to editorial changes in the manuscript, read and approved the final manuscript.

## Conflict of Interest

The authors declare that the research was conducted in the absence of any commercial or financial relationships that could be construed as a potential conflict of interest.

## Publisher’s Note

All claims expressed in this article are solely those of the authors and do not necessarily represent those of their affiliated organizations, or those of the publisher, the editors and the reviewers. Any product that may be evaluated in this article, or claim that may be made by its manufacturer, is not guaranteed or endorsed by the publisher.

## Footnotes

1
https://cancergenome.nih.gov/

2
http://www.ncbi.nlm.nih.gov/geo

## Supplementary Material

Supplementary Figure 1 Subgroup analysis of the survival of patients with different clinicopathological characteristics. **(A)** Age, **(B)** tumor status, and **(C)** FIGO stage.

Supplementary Figure 2 Kaplan–Meier (KM) survival curves of joint analysis of methylation and expression data in OC.

Supplementary Figure 3 Functional enrichment analysis of the differentially expressed genes (DEGs) between the high-risk and low-risk groups. **(A)** Top 10 most enriched Gene Ontology (GO) terms of these genes. **(B)** Top 20 most enriched pathways of these genes.

## Abbreviations

AKAP12: A kinase anchoring protein 12
AUC: area under curve
DEG: differentially expressed gene
DMG: differentially methylated gene
GO: Gene Ontology
KEGG: Kyoto Encyclopedia of Genes and Genomes
KM: Kaplan–Meier
PCA: principal component analysis
OC: ovarian cancer
GEO: Gene Expression Omnibus
GTEx: Genotype-Tissue Expression
OS: overall survival
ROC: receiver operating characteristic
TCGA: The Cancer Genome Atlas
TSG: tumor suppressor gene.
