# Corrigendum: Development and validation of a prognostic nomogram based on DNA methylation-driven genes for patients with ovarian cancer

**DOI:** 10.3389/fgene.2022.951409

**Published:** 2022-08-15

**Authors:** Min Zhou, Shasha Hong, Bingshu Li, Cheng Liu, Ming Hu, Jie Min, Jianming Tang, Li Hong

**Affiliations:** Department of Gynecology and Obstetrics, Renmin Hospital of Wuhan University, Wuhan, Hubei, China

**Keywords:** ovarian cancer, methylation, CpG sites, model, overall survival, biomarkers

In the original article, there was a mistake in Table 1 as published. In Table 1, we erroneously displayed the sample number and clinical data (age, grade and stage) of GSE49997 (*n* = 193) dataset as the sample number and clinical data of GSE26712 (*n* = 185) dataset. In fact, no clinical data is provided for the GSE26712 dataset, which only provides survival data. The corrected Table 1 appears below.

**TABLE 1:** Clinicopathologic characteristics of ovarian cancer (OC) patients in The Cancer Genome Atlas (TCGA) and Gene Expression Omnibus (GEO) cohorts.

Variables	TCGA cohort	GSE9891
	(*n* = 358)	(*n* = 273)
	N (%)	N (%)
Age (M ± SD, years)	59.4 ± 11.39	59.60 ± 10.55
Tumor size (M ± SD, cm)	0.90 ± 0.40	—
Grade		
1 and 2	43 (12.0)	112 (41.1)
3 and 4	315 (88.0)	161 (58.9)
Tumor status		
Tumor free	80 (22.3)	—
With tumor	236 (65.8)	—
Unknown	42 (11.7)	—
Lymphatic invasion		
No	46 (12.8)	
Yes	97 (27.1)	
Unknown	215 (60.1)	
Venous invasion		
No	38 (10.6)	—
Yes	60 (16.8)	—
Unknown	260 (72.6)	—
Stage		
I–II	20 (5.6)	41 (15.0)
III	284 (79.3)	209 (76.6)
IV	54 (15.1)	23 (8.4)
Primary therapy outcome		
Complete remission/response	202 (56.4)	—
Partial remission/response	42 (11.7)	—
Stable disease	21 (5.9)	—
Progressive disease	25 (7.0)	—
Unknown	68 (19.0)	—

In the original article, there was an error. GSE26712 dataset contains 185 patients instead of 193.

A correction has been made to **Dataset acquisition and pre-processing:**

“The DNA methylation data of OC were downloaded from TCGA^**1**^ database. The mRNA expression profiles of normal ovarianand OC samples were downloaded from the GTEx and TCGA databases using the University of California Santa Cruz (UCSC) Xena browser **(Chang et al., 2019**). In addition, the microarray data of GSE9891 and GSE26712 were acquired from GEO^2^ to represent independent cohorts of OC. Patients without survival time or status were excluded from the study. To ensure that the established prognostic signature had better generalization, TCGA dataset was used as the training set, and GSE9891 and GSE26712 datasets were used as the validation set. Cases without a certain age, FIGO stage, and tumor grade were excluded. Finally, 358 OC patients were included in TCGA set, 273 patients in the GSE9891 set, and 185 patients in the GSE26712 set. Table 1 lists the clinical features of the patients in the training and validation sets.”

In the original article, there was a mistake in Figure 1A as published. GSE26712 dataset contains 185 patients instead of 193. The corrected Figure 1A appears below.

**FIGURE 1:**
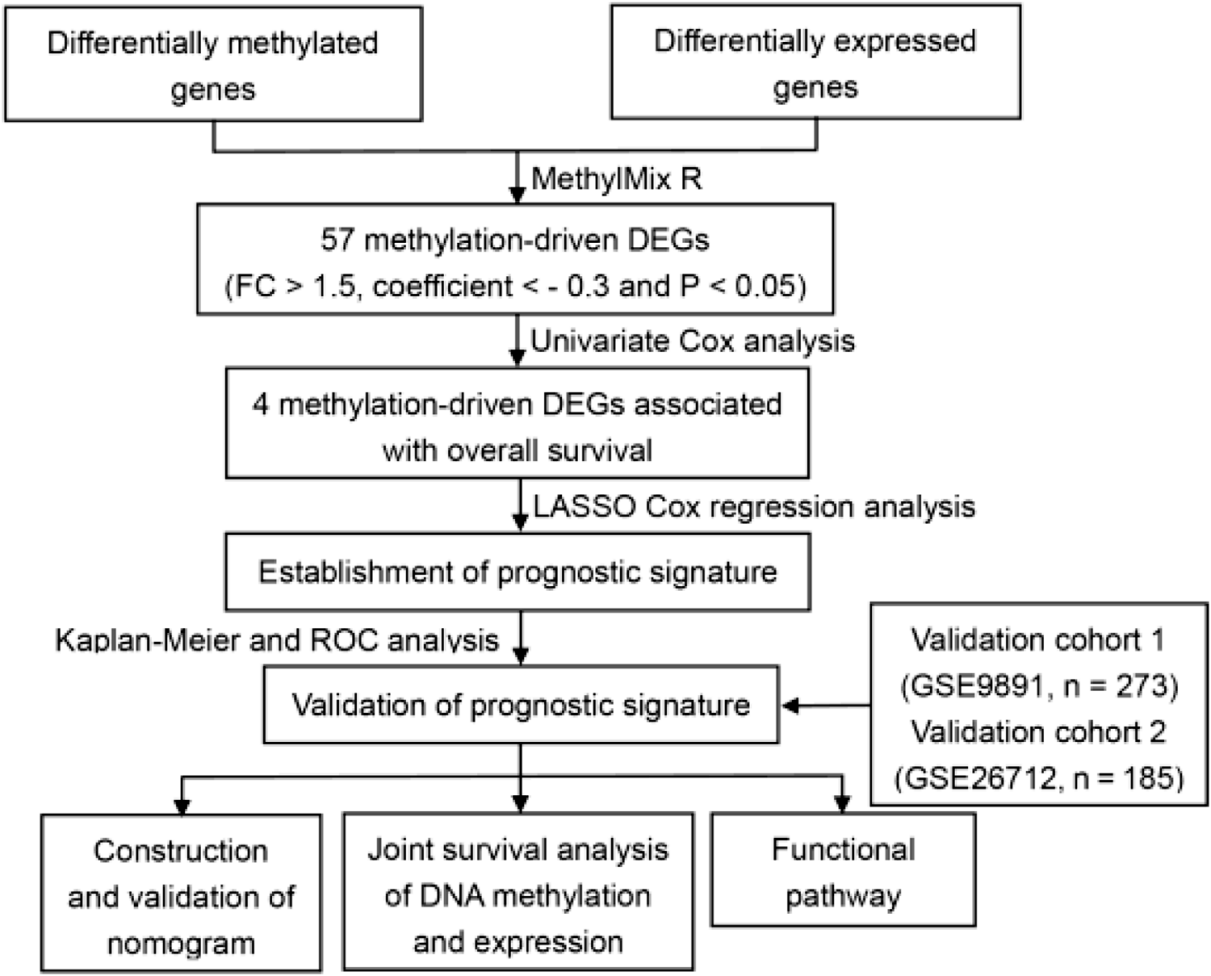
Identification of methylated related genes and flowchart of the establishment of novel prognostic signature. **(A)** The flowchart of the establishment of novel prognostic risk model for patients with ovarian cancer (OC). **(B)** The heatmap plot of 57 methylation related differentially expressed genes (DEGs) in OC. The color change from blue to red in the heatmap illustrates the trend from low to high methylation.

The authors apologize for these errors and state that this does not change the scientific conclusions of the article in any way. The original article has been updated.

