# Supplementary figures and images for: Development and Validation of a Prognostic Nomogram Based on DNA Methylation-Driven Genes for Patients With Ovarian Cancer
Source: Front Genet. 2021 Sep 9;12:675197. doi: 10.3389/fgene.2021.675197 (PMC8458765; doi:10.3389/fgene.2021.675197)

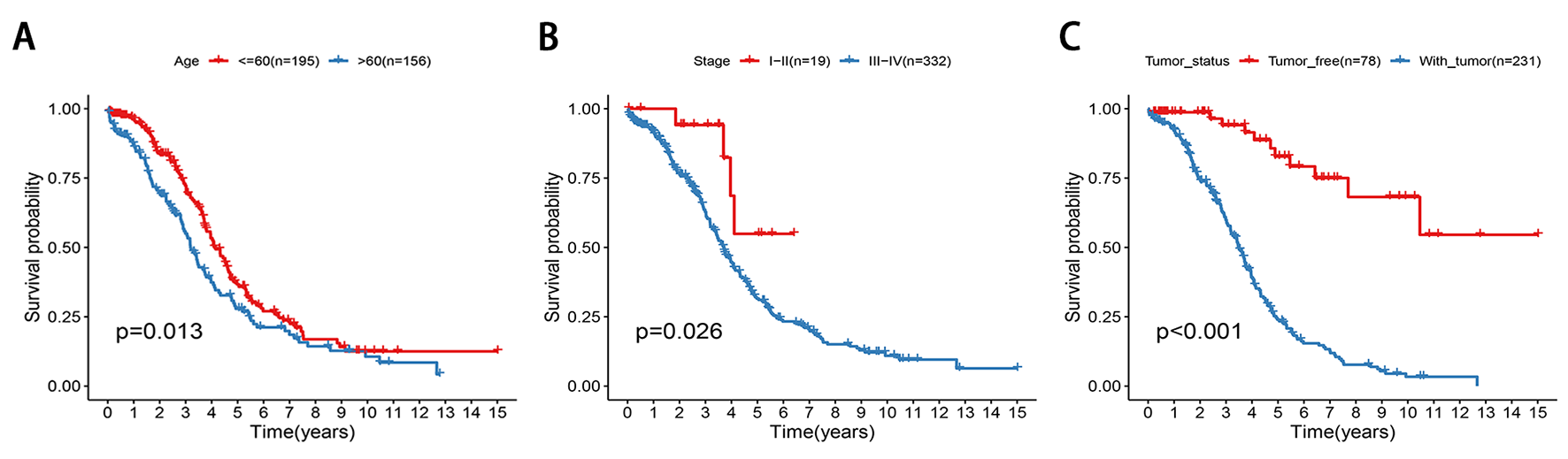

Supplement: Supplementary Figure 1 — Subgroup analysis of the survival of patients with different clinicopathological characteristics. (A) Age, (B) tumor status, and (C) FIGO stage. [file Image_1.TIF]

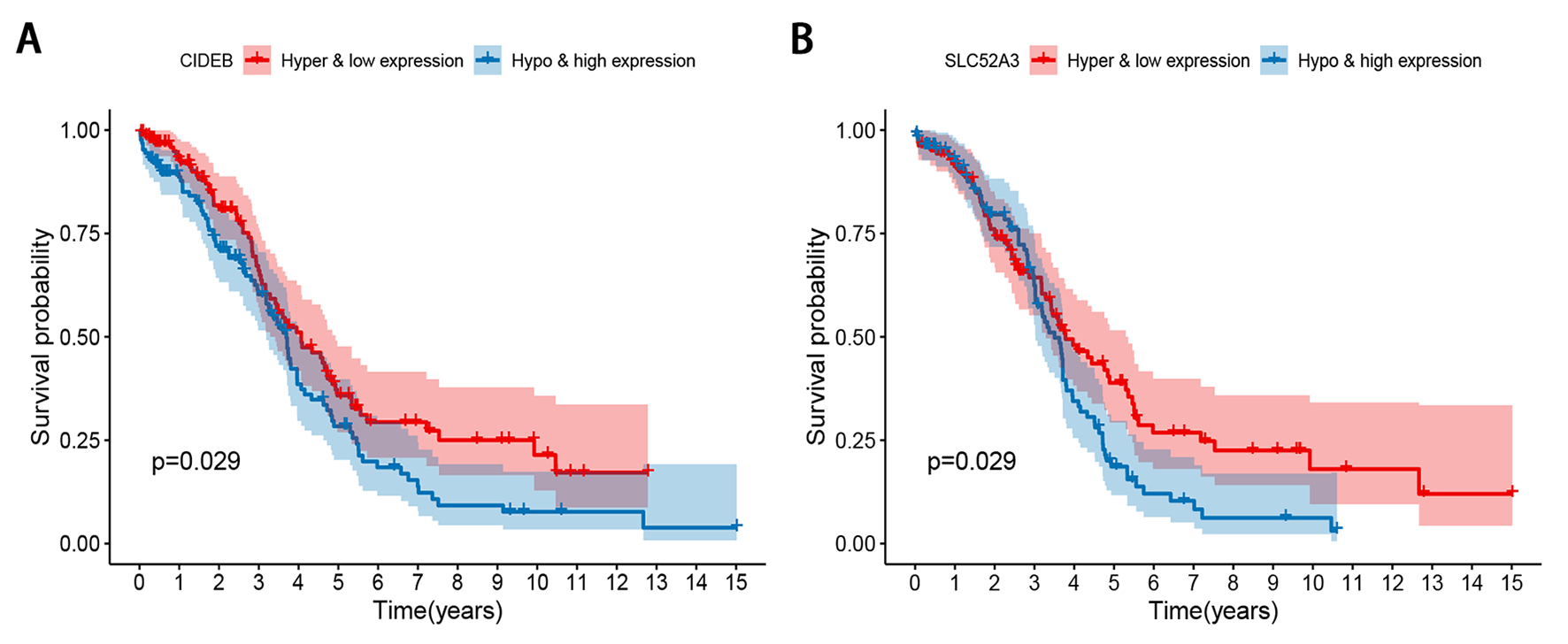

Supplement: Supplementary Figure 2 — Kaplan–Meier (KM) survival curves of joint analysis of methylation and expression data in OC. [file Image_2.TIF]

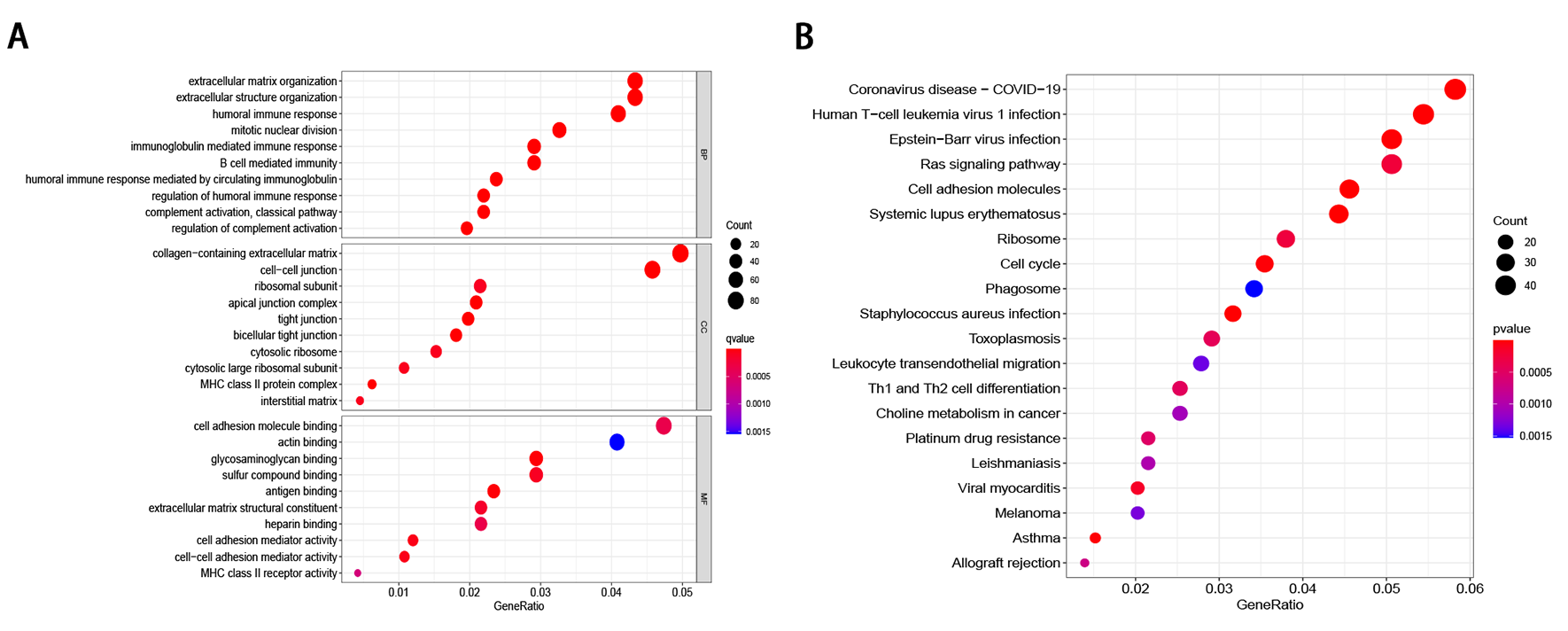

Supplement: Supplementary Figure 3 — Functional enrichment analysis of the differentially expressed genes (DEGs) between the high-risk and low-risk groups. (A) Top 10 most enriched Gene Ontology (GO) terms of these genes. (B) Top 20 most enriched pathways of these genes. [file Image_3.TIF]

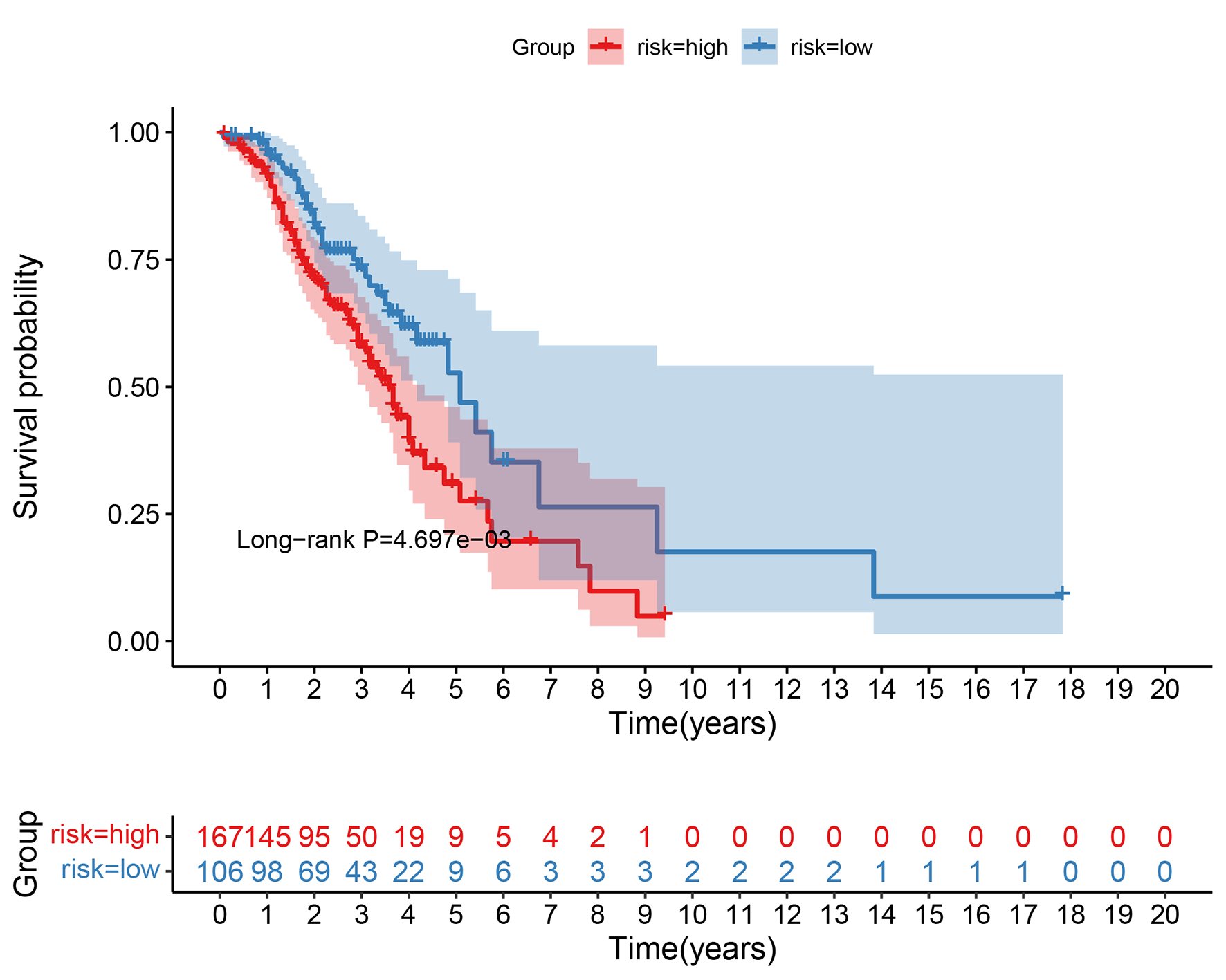

Supplement: Supplementary file 4 [file Image_4.TIF]

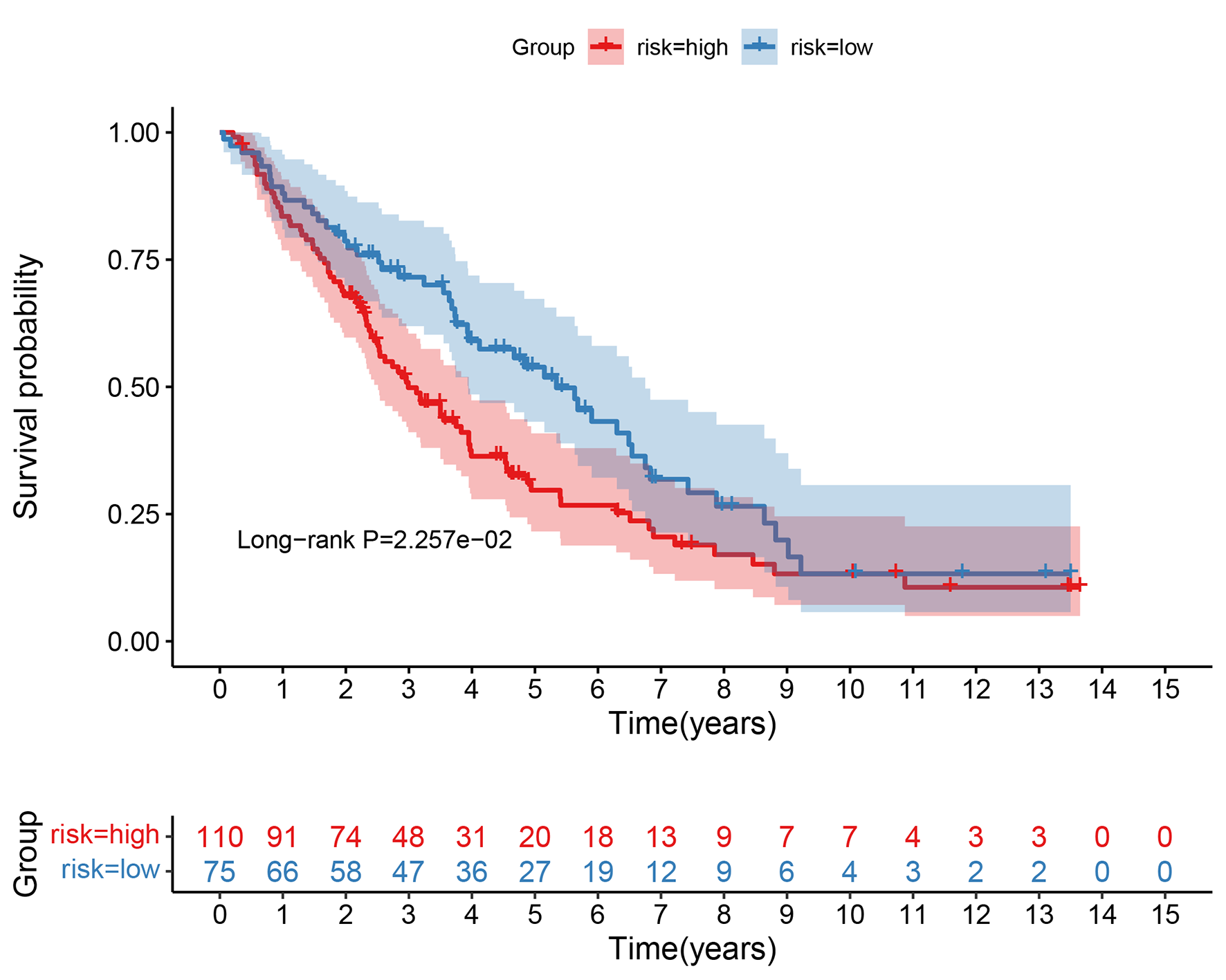

Supplement: Supplementary file 5 [file Image_5.TIF]
